# Maternal anthropometric variables and clinical factors shape neonatal microbiome

**DOI:** 10.1038/s41598-022-06792-6

**Published:** 2022-02-21

**Authors:** Riccardo Farinella, Cosmeri Rizzato, Daria Bottai, Alice Bedini, Federica Gemignani, Stefano Landi, Giulia Peduzzi, Sara Rosati, Antonella Lupetti, Armando Cuttano, Francesca Moscuzza, Cristina Tuoni, Luca Filippi, Massimiliano Ciantelli, Arianna Tavanti, Daniele Campa

**Affiliations:** 1 Department of Biology, University of Pisa, Pisa, Italy; 2 Department of Translational Research and of New Technologies in Medicine and Surgery, University of Pisa, Via San Zeno 37, 56127 Pisa, Italy; 3 Division of Neonatology, Santa Chiara Hospital, Pisa, Italy; 4 Centro Di Formazione e Simulazione Neonatale “NINA”, Azienda Ospedaliero-Universitaria Pisana, Pisa, Italy; 5 Neonatology and Neonatal Intensive Care Unit, Department of Clinical and Experimental Medicine, University of Pisa, Pisa, Italy

**Keywords:** Metagenomics, Microbiome, Next-generation sequencing

## Abstract

Recent studies indicate the existence of a complex microbiome in the meconium of newborns that plays a key role in regulating many host health-related conditions. However, a high variability between studies has been observed so far. In the present study, the meconium microbiome composition and the predicted microbial metabolic pathways were analysed in a consecutive cohort of 96 full-term newborns. The effect of maternal epidemiological variables on meconium diversity was analysed using regression analysis and PERMANOVA. Meconium microbiome composition mainly included Proteobacteria (30.95%), Bacteroidetes (23.17%) and Firmicutes (17.13%), while for predicted metabolic pathways, the most abundant genes belonged to the class “metabolism”. We observed a significant effect of maternal Rh factor on Shannon and Inverse Simpson indexes (p = 0.045 and p = 0.049 respectively) and a significant effect of delivery mode and maternal antibiotic exposure on Jaccard and Bray–Curtis dissimilarities (p = 0.001 and 0.002 respectively), while gestational age was associated with observed richness and Shannon indexes (p = 0.018 and 0.037 respectively), and Jaccard and Bray–Curtis dissimilarities (p = 0.014 and 0.013 respectively). The association involving maternal Rh phenotype suggests a role for host genetics in shaping meconium microbiome prior to the exposition to the most well-known environmental variables, which will influence microbiome maturation in the newborn.

## Introduction

Until recently, it was believed that the fetus develops in a completely sterile environment^1^. Therefore, microbial colonization of the newborn gastrointestinal tract was thought to begin at birth, through the exposure to microorganisms deriving from maternal vaginal or skin bacteria^2^, and from environmental sources such as the type of feeding (breastfeeding or artificial)^3^. However, recent technological advancements in the field of metagenomics based on Next Generation Sequencing (NGS) allowed the identification of microbial DNA in the placenta^4,5^, in the amniotic fluid^6,7^ and in the meconium^1,8^, suggesting the hypothesis that microbial colonization could occur in utero^7^.

Meconium is the first excretion product of newborn mammals, and it mainly consists of bile acids, pancreatic secretions, epithelial cells, and the residue of swallowed amniotic fluid^9^. Despite the fact that bacterial presence in meconium samples has been confirmed by several studies^8,10^, it is still unknown how early gut microbiome develops and which are the potential sources of maternal microorganism contributing to the fetal gut microbiome development^1^. It is believed that the maternal gut^1^, oral^4^, vaginal^11^ and uterine microbiomes^12^ are key sources for meconium microbiome development. However, it is still not completely clear how maternal microorganisms can reach the fetal gut and colonize it.

The composition of the meconium microbiome is variable among newborns and since the gut microbiome is an important regulator for a plethora of developmental physiological processes^13,14^, alterations of the microbiome composition during the prenatal, perinatal and neonatal stages can lead to short- and long-term consequences^15^, such as inflammatory bowel disease^16^, asthma^17^, allergies^18^ and altered immunological development^19,20^, and central nervous system disorders^21^. Despite its suggested involvement in short- and long-term health conditions, the actual knowledge on meconium microbiome is still limited and many fundamental questions still lack clear answers, such as which factors could influence its composition and diversity^11^.

Since the fetus is exposed to a limited number of stimuli and its development occurs in a relatively homogeneous and stable environment, the neonatal microbiome is characterized by a minor diversity than in adults^22^. Nonetheless, some factors are thought to influence the microbiome during the prenatal period or during delivery, shaping the microbiome in terms of both abundance and composition^2^. Among these factors the most investigated are maternal diet^23^, maternal stress^24^, maternal antibiotic exposure during pregnancy^25,26^, delivery mode^11^ and gestational age^27^. However, contrasting results have been observed in different studies, probably due to the small average sample size and to different statistical methods employed^28^.

In addition, despite increasing evidence on host genetics role in shaping the adult gut microbiome^29^, only one small study, conducted on twins, considered the potential effects of genetics on the meconium microbiome diversity and composition^30^. The aim of this study was to explore the meconium microbiome composition and the effect of maternal epidemiological factors on alpha and beta diversity indexes, in a homogeneous cohort of consecutively collected newborns.

## Results

### Study population

The study population consisted of 96 subjects, 42 females and 54 males, with a mean gestational age of 39.74 ± 1.18 and 39.58 ± 1.06 weeks, respectively (Supplementary figure SF1). The number of vaginally delivered (VD) and caesarean-section delivered (CSD) newborns was respectively 54 and 42.

Delivery mode and maternal antibiotic exposure were strongly associated (Fisher’s exact test p-value < 2*10^–16^).

All relevant information of the population in study are reported in Table 1.

**Table 1:** Epidemiological variables of the population in study.

Variables	N^a^	Mean	Standard deviation
Gestational age (weeks)	96	39.65	1.16
Maternal age (years)	96	34.72	5.25
Gravidic weight increase (kilograms)	93	12.66	4.32
Sex	Male: 54 Female:42	–	–
Maternal Rh factor	Positive: 85 Negative: 10	–	–
Maternal diabetes	Yes: 24 No: 72	–	–
Maternal antibiotic exposure	Untreated : 46 Treated: 49	–	–
Delivery mode^b^	CSD : 42 VD: 54	–	–

a—refers to the number of couples mother-newborn for which data were available for each epidemiological variable; b—“CSD” stands for caesarean delivery, while “VD stands for vaginal delivery.

### Sequencing and taxonomical assignment

Prokaryotic DNA was extracted and amplified in all 96 first pass meconium samples, and no DNA amplification was observed in PCR negative controls. Sequencing produced an average of 12,420 reads per sample, (standard deviation of 5546), of which a total of 5913 different 16S Amplicon Sequence Variants (ASVs) were identified. The analysis of meconium microbial composition was restricted from phylum to genus since the percentages of taxonomical assignments were greater than 75% in these taxonomical groups.

Bacterial domain consisted in most of the meconium microbiome. In fact, the average relative abundance of bacterial was 99.43%, ranging from a maximum of 100% (for 51 samples) to a minimum of 94.86%. Focusing only on the univocally assigned reads, the following numbers of taxa were obtained: 31 bacterial and 3 archaeal phyla; 65 bacterial and 5 archaeal classes; 187 bacterial and 10 archaeal orders; 291 bacterial and 7 archaeal families; 449 bacterial and 7 archaeal genera. A brief description of the Archaea taxa detected in meconium samples is reported in the following paragraph while the most common and abundant bacterial taxa are reported in the “Core microbiome” paragraph.

For Archaea, DNA was detected in 45/96 samples. The Nanoarchaeaeota phylum dominated the archaeal microbiome component with a prevalence of 100% and a relative abundance of 98.64 ± 5.68%, while Diapherotrites and Euryarchaeota phyla were only detected in two samples each, with relative abundances of 0.54% and 0.82% respectively. At the genus level, the dominant taxon was *Candidatus Pacearchaeota archaeon CG1_02_32_21* (prevalence of 15/45 samples and relative abundance of 21.50 ± 52.71%), followed by *Candidatus Pacearchaeota archaeon RBG_19FT_COMBO_34_9* (prevalence of 4/45, relative abundance of 4.38 ± 21.26%). Five taxa were detected in only one sample (*archaeon GW2011_AR10*, *Methanobacterium, Candidatus Diapherotrites archaeon ADurb.Bin253, Candidatus Pacearchaeota archaeon CG1_02_31_27* and *Nanoarchaeota archaeon SCGC AAA011-D5* with relative abundances of 0.08%, 0.73%, 0.36%, 0.42% and 0.21% respectively), while the remaining taxa, which made about the 72.33 ± 54.48% of archaeal microbiome in terms of abundance, resulted unclassified.

### Core microbiome

Seven taxa were selected at phylum level, corresponding on average to the 97.07 ± 2.25% of the entire microbiome in terms of relative abundance and ranging from a minimum of 90% to a maximum of 100% (for 12 samples). The Bacterial microbiome composition was mainly comprised of Proteobacteria (30.95 ± 23.52%), Bacteroidetes (23.17 ± 14.21%), Firmicutes (17.13 ± 25.87%), Patescibacteria (16.47 ± 13.42%), Actinobacteria (7.26 ± 7.84%), Verrucomicrobia (1.16 ± 1.76%) and Planctomycetes (0.94 ± 1.27%). Descending along the taxonomical classification to genus level, meconium microbiome core was composed of nine bacterial genera that made up about the 42.37 ± 22.93% of bacterial meconium microbiome. These were *Flavobacterium* (16.21 ± 12.54%) *Escherichia-Shigella* (14.11 ± 27.34%), *Staphylococcus* (5.13 ± 15.38%), *Streptococcus* (1.76 ± 5.88%), *Acinetobacter* (1.55 ± 3.33%), *Corynebacterium 1* (1.11 ± 2.06%), *Fluviicola* (0.85 ± 1.18%), *Limnohabitans* (0.83 ± 11.17%) and *Cutibacterium* (0.82 ± 1.57%). Detailed information on the core microbiome composition from phylum to genus level is reported in Supplementary table ST1.

### Metabolic pathways prediction

A total of 367 microbial pathways were recognized in the KEGG Orthology database. At the third level of classification six pathways were identified, with the most abundant labelled as “metabolism”, which had a relative abundance of 71.76 ± 16.36%, followed by “environmental information processing” (10.63 ± 20.77%) and “cellular processes” (5.49 ± 7.94%). The second level of classification identified 48 pathways with “carbohydrate metabolism” as the most frequent (relative abundance = 10.62 ± 17.83%), followed by “membrane transport” (6.43 ± 21.07%) and “signal transduction” (4.19 ± 5.68%). Each of these three metabolic categories contained several metabolic functions. In particular, 15 pathways belonged to “carbohydrates metabolism”, 25 to the “signal transduction”, and three to the “membrane transport” categories. The most abundant functions related to carbohydrate metabolism were “amino sugar and nucleotide sugar metabolism” (1.24 ± 0.49), “pyruvate metabolism” (1.18 ± 0.12) and “glycolysis” (1.12 ± 0.29). Among the most abundant pathways involved in signal transduction there were “two-component system” (3.76 ± 0.61), “HIF-1 signaling pathway” (0.10 ± 0.03) and “MAPK signaling pathway” (0.1 ± 0.02), while only the three pathways “ABC transporters” (4.68 ± 0.89), “phosphotransferase system” (1.19 ± 1.35) and “bacterial secretion system”(0.57 ± 0.14) belonged to the “membrane transport” category. The complete list of the 367 metabolic pathways is reported in the Supplementary table ST2.

A general homogeneity was observed in terms of pathway composition among samples as reported in Supplementary figures SF2 and SF3. This was confirmed by comparing the Pielou’s evenness index calculated on both taxa and predicted metabolic pathways. The latter had a statistically significant higher mean (p-value = 1.794 × 10^–6^) and a minor interquartile range.

### Maternal contribution to meconium alpha diversity

Gestational age resulted significantly associated with observed richness and Shannon index (p-values of 0.018 and 0.037 respectively), while a borderline effect was observed on Inverse Simpson index (p-value = 0.075). The three estimated beta coefficients were all negative, indicating a reduction in microbial diversity with the increasing gestational age.

Maternal diabetic status showed a borderline association with all three diversity indexes, with p-values of 0.086, 0.091 and 0.065 for observed richness, Shannon and Inverse Simpson indexes, respectively. The negative estimated beta coefficients suggested a trend for which newborns delivered by diabetic mothers had a lower alpha diversity than newborns from non-diabetic mothers.

**Figure 1:**
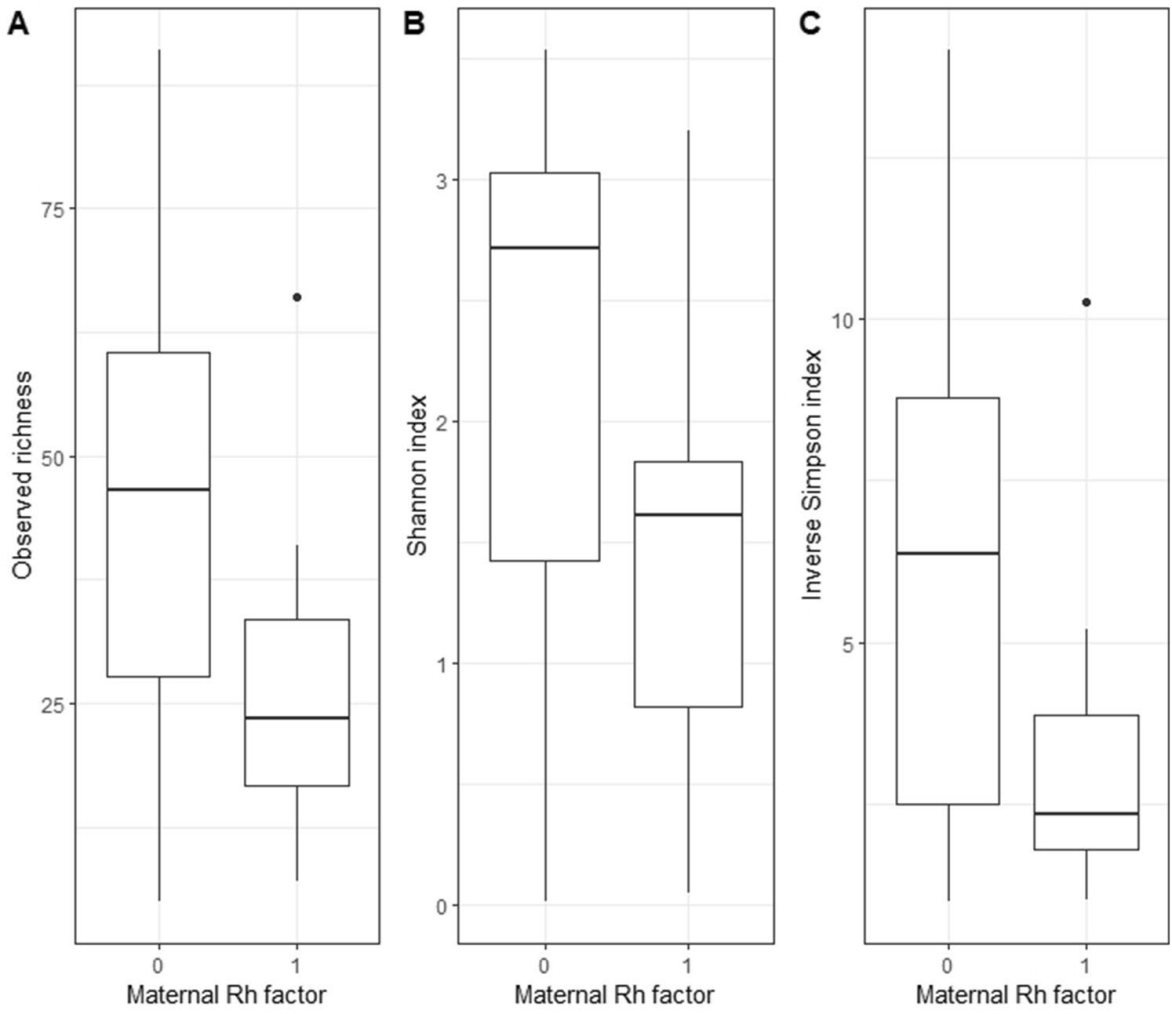
Box plots of alpha diversity by maternal Rh factor. The three panels (**a**), (**b**) and (**c**) report the observed richness index, Shannon index and Inverse Simpson index respectively.

Newborns having Rh negative mothers showed an alpha diversity reduction compared to newborns from Rh positive mothers, with p-values of 0.045 and 0.049 for Shannon and Inverse Simpson index respectively. For observed richness, a borderline association was observed (p-value = 0.078). All the results are reported in Fig. 1 and Table 2. Following the investigation at the taxonomical level, seven microbial genera had higher abundances in Rh positive samples: *Pseudarcicella* (0.25 ± 0.45%), *Acinetobacter* (1.56 ± 3.34%), *Polynucleobacter* (0.30 ± 0.46%), *Cutibacterium* (0.83 ± 1.58%), *Enterococcus* (7.40 ± 22.40%), *Paracoccus* (0.78 ± 3.75%) and *Bacteroides* (2.97 ± 8.84%). However, none of their distributions remained statistically different after multiple testing correction. The box plots are reported in the Supplementary figure SF4.

**Table 2:** Association between clinical and anthropometric variables and alpha diversity indexes.

Independent variable	Outcome					
	Observed Richness		Shannon index		Inverse Simpson index	
	Coeff. (95%CI)	p-value	Coeff. (95%CI)	p-value	Coeff. (95%CI)	p-value
Delivery mode	6.823(−1.805–15.451)	0.125	0.285(−0.096–0.665)	0.143	1.074(−0.316–2.464)	0.130
Maternal diabetics status^a^	−8.546(−18.307–1.215)	0.086	−0.371(−1.018–0.433)	0.091	−1.476(−3.044–0.092)	0.065
Sex^a^	2.950(−5.558–11.459)	0.498	0.157(−0.217–0.531)	0.411	0.370(−1.002–1.742)	0.597
Gestational age^a^	−4.270(−7.805–0.736)	**0.018**	−0.167(−0.323–0.010)	**0.037**	−0.524(−1.100–0.052)	0.075
Maternal age^a^	−0.558(−1.358–0.242)	0.175	−0.002(−0.033–0.037)	0.918	0.025(−0.105–0.155)	0.704
Gravidic weight increase^a^	0.165(−0.847–1.176)	0.750	0.009(−0.036–0.053)	0.703	0.023(−0.140–0.186)	0.782
Maternal antibiotic exposure	3.072(−5.705–11.849)	0.494	0.174(−0.210–0.558)	0.377	0.623(−0.788–2.034)	0.389
Maternal Rh factor^a^	−8.503(−18.137–1.130)	0.078	−0.571(−1.130–0.011)	**0.045**	−1.688(−3.372–0.004)	**0.049**

All analyses were adjusted for the DNA extraction batch used.

Significant values are in bold.

### Maternal contribution to meconium beta diversity

**Figure 2:**
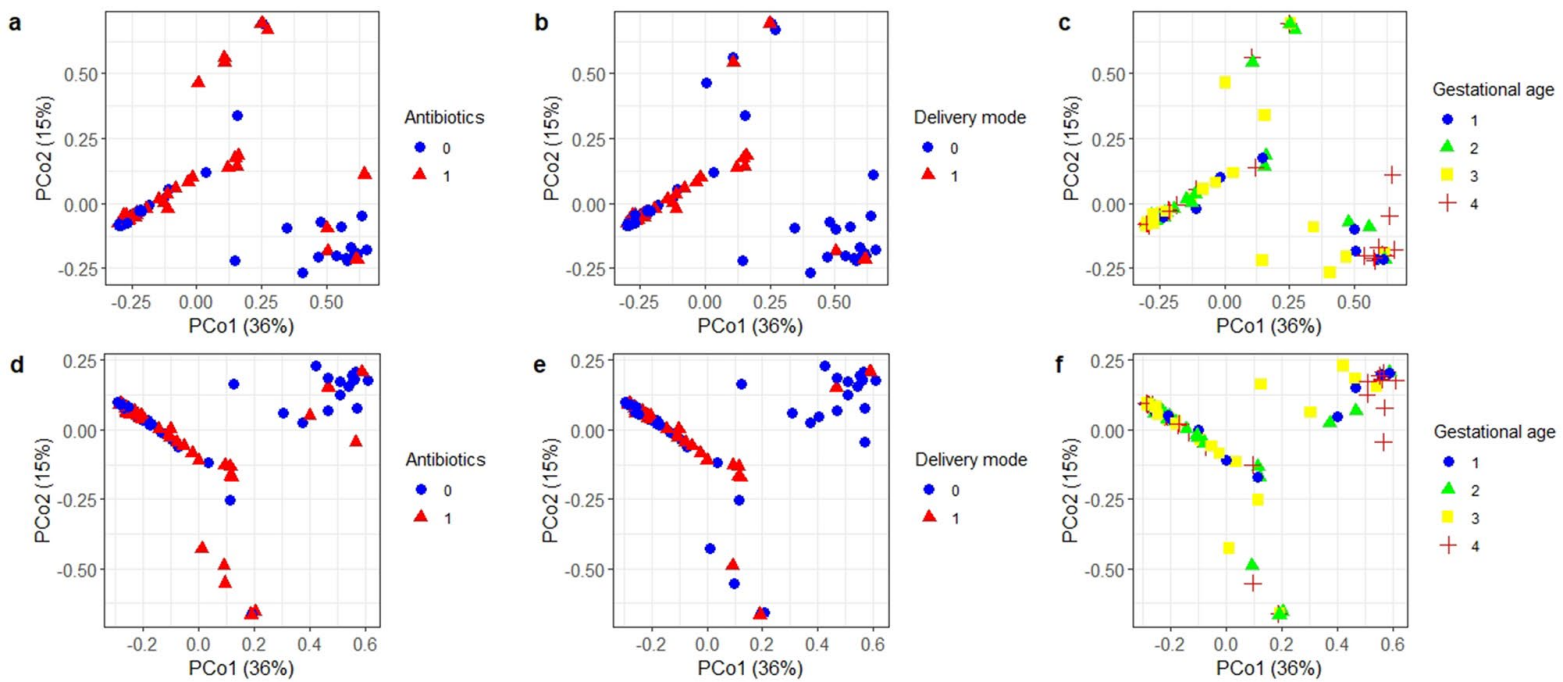
Ordination plot for the first two principal coordinates based on Bray–Curtis (upper plots) and Jaccard (lower plots) dissimilarities reported by, from left to right, maternal antibiotic exposure (**a**, **d**), delivery mode (**b**, **e**) and gestational age (**c**, **f**). For simplicity of graphical representation, gestational age is reported using quartiles.

The PCoA plot of Bray–Curtis and Jaccard dissimilarities indexes showed a tendency towards clustering by delivery mode and maternal antibiotic exposure (Fig. 2). The PERMANOVA test detected a significant effect of delivery mode, maternal antibiotic exposure and gestational age on both indexes as reported in Table 3 and Fig. 2. None of the other variables had a significant effect on neither index. The fraction of variance explained by delivery mode, antibiotic exposure and gestational age was generally low for both dissimilarity measures, ranging from a minimum R2 of 2.5% to a maximum R2 of 5.1% (Table 3). A significant effect of antibiotic exposure on both Bray–Curtis and Jaccard dissimilarities was detected even when restricting the sample to VD newborns only (R2 = 0.041, p-value = 0.044; R2 = 0.035, p-value = 0.048 respectively). Additionally, both delivery mode and antibiotic exposure were significant when included in the same PERMANOVA sequential test, while they explained a different proportion of variance (Table 4). The significance of marginal effects for each of the two variables was further confirmed by distance-based redundancy analysis, after controlling in turn for the other variable (p-values of 0.012 and 0.031 for delivery mode and antibiotic exposure using Bray–Curtis, and 0.014 and 0.025 using Jaccard dissimilarity respectively).

**Table 3:** Results of the PERMANOVA test using Bray–Curtis and Jaccard dissimilarities.

	Bray–Curtis		Jaccard	
	R2	p-value	R2	p-value
Sex^a^	0.011	0.322	0.01	0.369
Maternal diabetes^a^	0.009	0.436	0.009	0.469
Gestational age^a^	0.030	**0.013**	0.025	**0.014**
Delivery mode^a^	0.051	**0.001**	0.037	**0.001**
Maternal age^a^	0.007	0.678	0.007	0.748
Gravidic weight increase^a^	0.005	0.858	0.007	0.807
Maternal antibiotic exposure	0.044	**0.002**	0.034	**0.002**
Maternal Rh factor^a^	0.018	0.143	0.015	0.155

Significant values are in bold.

^a^The sequential test was done adjusting for the used DNA extraction batch.

**Table 4:** Results of the PERMANOVA test using both delivery mode and antibiotic exposure.

First sequential test	Bray–Curtis		Jaccard		Second sequential test	Bray–Curtis		Jaccard	
	R2	p-value	R2	p-value		R2	p-value	R2	p-value
Maternal antibiotic exposure	0.044	**0.001**	0.034	**0.001**	Delivery mode	0.5	**0.002**	0.037	**0.001**
Delivery mode	0.028	**0.01**	0.022	**0.014**	Maternal antibiotic exposure	0.022	**0.025**	0.019	**0.022**

Both sequential tests were adjusted for DNA extraction batch as first covariate. In the first test, maternal antibiotic exposure was added as covariate prior to delivery mode, while in the second test their order was inverted.

Significant values are in bold.

When including the three significant variables in the same PERMANOVA model, gestational age was not significant unless it was introduced in the model prior to the other two covariates.

## Discussion

Sequencing results indicated that the Bacteria domain represented the vast majority of the meconium microbiome. Most microorganisms were however rare, with a prevalence of less than 10%, so a microbial core defined as the overall taxa with a prevalence of at least 50% was determined at each taxonomical level to identify the most prevalent prokaryotic microorganisms.

The first bacterial colonizers of gut microbiome are thought to be facultative anaerobes, like *Escherichia-Shigella* species^1^, which as aerotolerant microorganisms, are believed to be implicated in the oxygen’s level reduction into the intestine, shaping the neonatal gut environment and contributing to the successive proliferation of anaerobic bacteria^31^. Accordingly, in our study the genus *Escherichia-Shigella* was observed as one of the most common one (78/96, 81.3%), and one of the most abundant (relative abundance of 14.11%). The findings of this research are consistent with previous studies^22,32,33^.

Few studies provided information on the archaeal component of meconium microbiome^32^, even though the metabolic importance of some archaeal taxa, such as gut methanogens, has been already reported in adult humans^34,35^. Accordingly to a recent work by Wampach and colleagues^32^, our results indicated a very low archaeal contribution to the microbial composition and diversity in terms of both abundance and prevalence. Notably, most of the archaeal taxa resulted as taxonomically unclassified, underlying the poor human knowledge of archaeal genomes, even though they are considered as ubiquitous members of the adult gastrointestinal tract^36^.

In addition to the taxa’s phylogenetic distributions, we assessed the composition of meconium samples in terms of predicted biochemical pathways. To the best of our knowledge, this is the larger study in terms of sample size after that of Dong and colleagues^37^ to characterize the metabolic potential of meconium prokaryotic community. Most predicted genes were involved in metabolic functions (about the 80% of 367 pathways), comprising both catabolism and anabolism. More precisely, carbohydrate metabolism was the most important component in terms of abundance, among the general category of metabolic functions. Despite the differences in the composition of the microbial community, the general homogeneity of pathways distribution among samples highlights the importance of function preservation, even if it may involve different bacteria. This discrepancy between pathways homogeneity and microbial heterogeneity is in agreement with previous observations^38,39^.

Since there is still a limited understanding of which factors affect meconium microbial composition, we investigated whether some of the most important maternal factors could impact microbial diversity. To express microbial diversity, we selected the most common within-samples diversity indexes (observed richness, Shannon and Inverse Simpson indexes) and the most common between-samples diversity measures (Bray–Curtis and Jaccard dissimilarities).

For alpha diversity, our results suggest an association between maternal Rh antigen phenotype (negative or positive) and alpha diversity, with a significant reduction in microbial diversity in newborns from Rh negative mothers compared to those from Rh positive mothers. Despite blood groups antigens were already known for their association with altered susceptibility to infections^40^ and were recently investigated in association studies in the adult^29,41,42^, this is the first study to report the association between the Rh phenotype and the microbial alpha diversity in meconium samples.

A recent study carried out in a very large population reported a genome-wide association between the *ABO* gene and two bacterial genera^29^. However, only adult individuals were enrolled, while in newborns this association still remains to be tested.

Since the ABO glycosylation pattern based on its phenotype has an effect on symbiotic bacterial adhesion^43,44^, it is worth noting in this context that the Rh complex offers several glycosylation sites^45^, which could be secondary binding targets for bacterial adhesion structures^46^.

During pregnancy the maternal intestinal barrier may be subjected to variations in permeability^47^. This event could facilitate maternal microbes in reaching the bloodstream or translocating into the lymphatic vessels to get to the placenta from the maternal gut^11^. Another mechanism for bacterial translocation has been proposed in an interesting study by Rescigno and colleagues, for which dendritic cells directly sample bacteria from the gut by using the dendritic processes^48^. After reaching the lymphatic system, bacteria could exploit circulating cells^6,49^ to reach different anatomical locations and colonization sites, such as the fetal gut^11^. The possibility for bacteria to use the bloodstream to move into the host was already described during pregnancy^50^, while it was reported in animal models that bacteria could reach the amniotic fluid from the gut^6^.

The association between Rh phenotype and alpha diversity suggests a role for genetic variation in determining the early composition and variability of the microbiome prior to the exposition to the post-natal environment. However, further investigations are needed to validate the association and address the molecular mechanism involved.

A significant effect of gestational age was observed on both alpha and beta diversity. The importance of gestational age as a conditioning factor for the neonatal gut microbiome progression and development has been highlighted in several studies^51,52^, but its contribution in explaining meconium diversity at birth only in term newborns has never been specifically investigated. In fact, the majority of the studies focused on the differences between term and preterm newborns, or between early- and late-preterm newborns^53,54^, while in our study all neonates were born at term. For beta diversity, we observed a significant effect of delivery mode on both Jaccard and Bray–Curtis dissimilarities. This is in line with the results of Wong and colleagues^25^, a study based on a comparable sample size (n = 106) and with other smaller-sized studies^33,53^. However, such result is in contrast with other reports, some of which based on larger sample sizes^37,55^ that however considered different beta diversity indexes, like phylogeny-based weighted and unweighted Unifrac distances- and other studies with smaller sample sizes^22,56^. So, the hypothesis for which the different newborn’s exposure during vaginal delivery or caesarean section leads to an altered microbial diversity and composition at birth is still a controversial aspect, and further studies are needed to fully address this point.

Antibiotic exposure can affect the microbial colonization in early life^57^ but less is known about the effects of antibiotic-based treatments on the first-meconium microbial content. In our study we found evidence for a potential effect of maternal antibiotic exposure on meconium beta diversity in vaginally-delivered newborns only, as well as in the whole sample, as confirmed in two recent studies^25,58^. Moreover, our results suggest that the effect of delivery mode on meconium microbiome within the first hours after birth is mainly (but not totally) due to the maternal antibiotic exposure preceding birth. Notably, this does not exclude an effect of delivery mode: basing on our findings, they both influence the meconium microbiome composition. However, since gestational age and delivery mode are not completely independent, there may be a complex interplay between delivery mode, maternal antibiotic exposure and gestational age. One of the main causes of between-studies variability in microbiome research is the absence of standardized protocols and pipelines. Therefore, different studies could introduce bias according to the type of methodology and statistical analysis used. In contrast with most of the studies analysing the meconium microbiome composition, we performed all analyses on non-rarefied data to avoid a decrease in sensitivity and the omission of valid data^59^. To control for contamination and correctly estimate community diversity, we used several positive and negative controls in PCR and all statistical analyses were adjusted for the batch used for DNA extraction, while a unique sequencing batch was used for all samples. However, meconium is a very complex matrix, which is characterized by a low biomass and by the contemporary presence of many PCR inhibitors. These conditions are considered important in the light of potential microbial contamination. Despite several precautions were taken to eliminate cross-contamination, we did not include negative controls for the extraction process. However, by adjusting all analyses by the DNA extraction batch used, we were able to avoid that potential microbial contaminants in the extraction reagents could significantly affect our results.

A potential limitation of our study lies in the methodological approach that we adopted: despite 16S amplification is a widely diffused technique for clinical microbial and microbiome studies, there may be some issues related to potential mutations occurring in the amplification procedure, that in our study was repeated twice. However, this potential bias can be limited by setting optimal PCR conditions for the amplification process and by removing chimeric sequences with bioinformatic tools. Anyway, a method that can completely remove artifacts of this kind still need to be developed^60,61^. Another limitation is the absence of maternal and environmental samples, whose analysis could have provide additional information on meconium microbial content. Despite this potential limitation, we obtained results very similar to those of other studies and thus it is unlikely that our estimates and inferences were totally driven by environmental contamination. In conclusion, our results suggest an association between maternal Rh factor and alpha diversity, and an effect of maternal antibiotic exposure and delivery mode on beta diversity, while gestational age resulted associated with both alpha and beta diversity of meconium. However, the fact that a large proportion of microbial diversity was essentially unexplained despite the use of most well-studied clinical and anthropometric variables^27^ clearly suggests that other factors may have a role in shaping meconium microbiome diversity. In particular, the interesting association between the genetically determined maternal Rh factor and alpha diversity indexes suggests that host genetics could have a role in newborn meconium microbiome composition, an intriguing finding that merits further characterization.

## Materials and methods

### Study population

A cohort of 96 newborns was enrolled between 2018 and 2020. All first pass meconium samples were collected after spontaneous expulsion in delivery room or within the first 24 h after birth, at the Neonatology Division of Santa Chiara Hospital of the Azienda Ospedaliera Universitaria Pisana AOUP. Each sample was collected from diaper using sterile swabs and put into sterile polypropylene tubes.

For each couple mother-newborn, anthropometric data (maternal age, gravidic weight increase) and clinical variables (gestational age, delivery mode, maternal Rhesus factor –Rh, maternal antibiotic exposure and maternal diabetic status) were collected.

Mothers in the VD group were treated with antibiotics in case of Group B *Streptococcus* detection in the birth canal and/or rectum, or in case of failed induction after more than 18 h since the rupture of the membranes. Prophylactic antibiotics were instead administered to all mothers in the CSD group prior to delivery.

All mothers were healthy subjects, with no diagnosis of chorioamnionitis, pre-eclampsia and/or eclampsia and no clinical diagnosis potentially impacting the meconium microbial community was reported at the Santa Chiara Hospital.

Only healthy term-newborns with a gestational age of at least 37 weeks and with a minimum Apgar score of 7 were included. In addition, a written informed consent was voluntarily subscribed by the parents of each newborn.

Exclusion criteria were preterm birth, suspected genetic syndrome or metabolic disease, an Apgar score lower than 7 and parents’ refusal to subscribe the informed consent.

The ethical committee of Meyer Pediatric Hospital in Florence, which is the elected IRB for all the pediatric studies in the Tuscany region of Italy, approved this study. The study was performed according to the ethical standards of the Declaration of Helsinki (1964).

### DNA extraction and amplification

Meconium samples were collected using sterile instruments and sterile lab equipment. After collection, meconium samples were stored at -20 °C. DNA extraction was performed using QIAamp Fast DNA Stool Mini Kit (QIAGEN). For sample amplification hot start PCR was performed on the V3-V4 285 bp sub-regions belonging to the 16S ribosomal gene, using the PRO341F and PRO805R set of universal primers for prokaryotic detection. Positive and negative controls were included in PCR reactions. For each sample, the extracted DNA was amplified using 0.2 µl Platinum Polymerase at a final concentration of 2U/reaction, in a reaction volume of 25 µl, which included Buffer without Mg^2+^(2.5 µl, 10x), MgSO_4_ (1 µl, 50 mM), dNTPs (0.5 µl, 5 mM), PRO341F (1 µl, 1 µM) and PRO805R (1 µl, 1 µM) primers. A standard thermic profile was used (1 cycle at 94˚C for 2’, 30 cycles at 94˚C for 30’’, 56˚C for 30’’ and 72˚C for 45’’ and 1 final cycle at 72˚C for 7’). Due to the low biomass of meconium samples, a second PCR round was repeated on the amplicons from the previous amplification step. Additional data on the amplification procedures are reported in Supplementary note. Then, amplicons were separated by electrophoretic runs at 120 V for 25 min in ethidium bromide 2% agarose gel and visualized at the BioDoc-It Imaging System (UVP, USA). Positive and negative controls were included during PCR reactions and electrophoretic runs. Additional details on the primer sequences are provided in Supplementary table ST3.

### DNA purification, sequencing and taxonomic assignment

Amplified DNA was checked by electrophoresis, as a prerequisite for the V3-V4 region sequence purification, which was performed by GeneAll Expin Combo GP (GeneAll Biotechnology) following the manufacturer’s instructions. Purified DNA quantification was carried out using NanoDrop Lite UV–Vis Spectrophotometer (TermoFisher Scientific). The average DNA concentration yield was 11.11 ± 7.33 ng/µl. Samples were then stored at -20˚C until sequencing.

Sequencing was performed using a unique batch for all samples on a Mi-Seq platform with a MiSeq kit by Bio-Fab Research (Bio Fab Research srl, Rome, Italy). Quality control on raw sequence data in fastq format was performed using FastQC v0.11.9, and dada2 plugin of QIIME2 (available at https://qiime2.org/) was used for denoising, merge and chimera detection, while for secondary analysis BBMap version 38.79 was used (https://sourceforge.net/projects/bbmap/).

Amplicon sequence variants (ASVs) were obtained as output. Then, taxonomic assignment based on 16S rRNA gene profiling was made using QIIME2 and SILVA v132 reference database (available at https://www.arb-silva.de/).

### Meconium core microbiome

In this study only taxa present in at least 50% of the samples were included in the analysis and their relative abundances were used to evaluate the composition of meconium microbiome at different taxonomical levels of classification. The 50% threshold chosen in this study is in line with recent data by Wang and colleagues, in which a similar sample size was analysed^62^. Taxa abundances were reported using mean ± standard deviation.

### Prediction of metabolic pathways

The pathways of metabolic activity of the meconium microbial community were predicted using Tax4Fun2 (downloaded as an R package: GitHub-bwemheu/Tax4Fun2), which is a recently developed bioinformatic tool that allows functional prediction from 16S gene sequences^63^. The algorithm blasts 16S fasta sequences to NCBI-RefSeq database, and then extracts metabolic and functional information from KEGG orthology database (https://www.genome.jp/kegg/ko.html). Contextually, using the abundance-related data, provided as an OTU-table, the algorithm computes the functional profile of each sample. The heterogeneity of metabolic composition was analysed and compared to that of the taxonomical composition using the Mann–Whitney-Wilcoxon rank sum test on Pielou’s evenness index^64^. Pathway abundances were reported using mean ± standard deviation.

### Association between clinical and anthropometric variables and alpha diversity

Observed richness, Shannon index and Inverse Simpson index were selected as alpha diversity measures^65^ and were calculated using R’s *vegan* package (https://CRAN.R-project.org/package=vegan).

Normalization of raw data was performed prior to alpha diversity indexes calculations by dividing reads counts by the corresponding sample size and multiplying by the size of the smaller sample^66^. The effect of the most relevant clinical and anthropometric factors –selected from literature^27^- and alpha diversity indexes was explored with a regression analysis using the generalized linear model. Gestational age, maternal age, gravidic weight increase, maternal Rh factor, maternal diabetic status, maternal antibiotic exposure, delivery mode and sex were used as independent variables, with the first three expressed as continuous variables and the others as binary factors, while diversity indexes were used as outcome variables. Even though all DNA samples were isolated with the same method, the analyses were adjusted also for DNA extraction kit batch used to avoid confounding bias.

Since an association was found between maternal Rh and alpha diversity, taxonomic differences by Rh factor were investigated applying the Wilcoxon rank sum test on all bacterial genera with a prevalence of at least a quarter of the sample size. P-values were adjusted using FDR correction (Benjamini–Hochberg method) to account for multiple comparison.

### Association between clinical and anthropometric variables and beta diversity

To measure beta diversity, Jaccard and Bray–Curtis dissimilarities^67^, were calculated using R’s *vegan* package (https://CRAN.R-project.org/package=vegan), while principal coordinates analysis (PCoA) was used to explore the association between the epidemiological variables listed above and beta diversity. To test the statistical significance the PERMANOVA test was employed using the R’s adonis function (*vegan* package) with 1000 permutations. All analysis were adjusted for DNA extraction batch used.

## Supplementary Information

Supplementary Information.

## Data Availability

The datasets generated and analysed during the current study will made available to researchers who submit a reasonable and detailed request to the corresponding author, conditional to approval of the Ethics Commission of the Meyer Children Hospital of Florence which is the appointed IRB for all the pediatric study in the Tuscany region. Data will be stripped from all information allowing identification of study participants. Sequencing data have been made publicly available through FASTQ files submission to Sequence Read Archive with project accession number PRJNA779839.

## Code availability

RStudio software version 4.0.3 (2020–10-10) was used for statistical analysis.
