# Supplementary material for: Maternal anthropometric variables and clinical factors shape neonatal microbiome
Source: Sci Rep. 2022 Feb 21;12:2875. doi: 10.1038/s41598-022-06792-6 (PMC8861021; doi:10.1038/s41598-022-06792-6)
Supplement: Supplementary file 1 — Supplementary Information. [file 41598_2022_6792_MOESM1_ESM.docx]

**Scientific reports**

**Maternal anthropometric variables and clinical factors shape neonatal microbiome**

Riccardo Farinella^1‡^, Cosmeri Rizzato^2‡*^, Daria Bottai^1^, Alice Bedini^3^, Federica Gemignani^1^, Stefano Landi^1^, Giulia Peduzzi^1^, Sara Rosati^3^, Antonella Lupetti^2^, Armando Cuttano^3,4^, Francesca Moscuzza^3^, Cristina Tuoni^3^, Luca Filippi^5^, Massimiliano Ciantelli^3,4#^, Arianna Tavanti^1#^, Daniele Campa^1#^

^1^ Department of Biology, University of Pisa, Pisa, Italy

^2^ Department of Translational Research and of New Technologies in Medicine and Surgery, University of Pisa, Pisa, Italy

^3^ Division of Neonatology – Santa Chiara Hospital – Pisa, Italy

^4^ Centro di Formazione e Simulazione Neonatale "NINA", Azienda Ospedaliero-Universitaria Pisana, Pisa, Italy.

^5^ Neonatology and Neonatal Intensive Care Unit, Department of Clinical and Experimental Medicine, University of Pisa, Italy.

**^‡^These authors share the first position**

**^#^ These authors share the last position**

**Corresponding author:**

Cosmeri Rizzato

Department of Translational Research and of New Technologies in Medicine and Surgery,

University of Pisa,

Via San Zeno 37,

56127 Pisa Italy

Tel +39 050 2213684

Fax +39 050 2213711

**
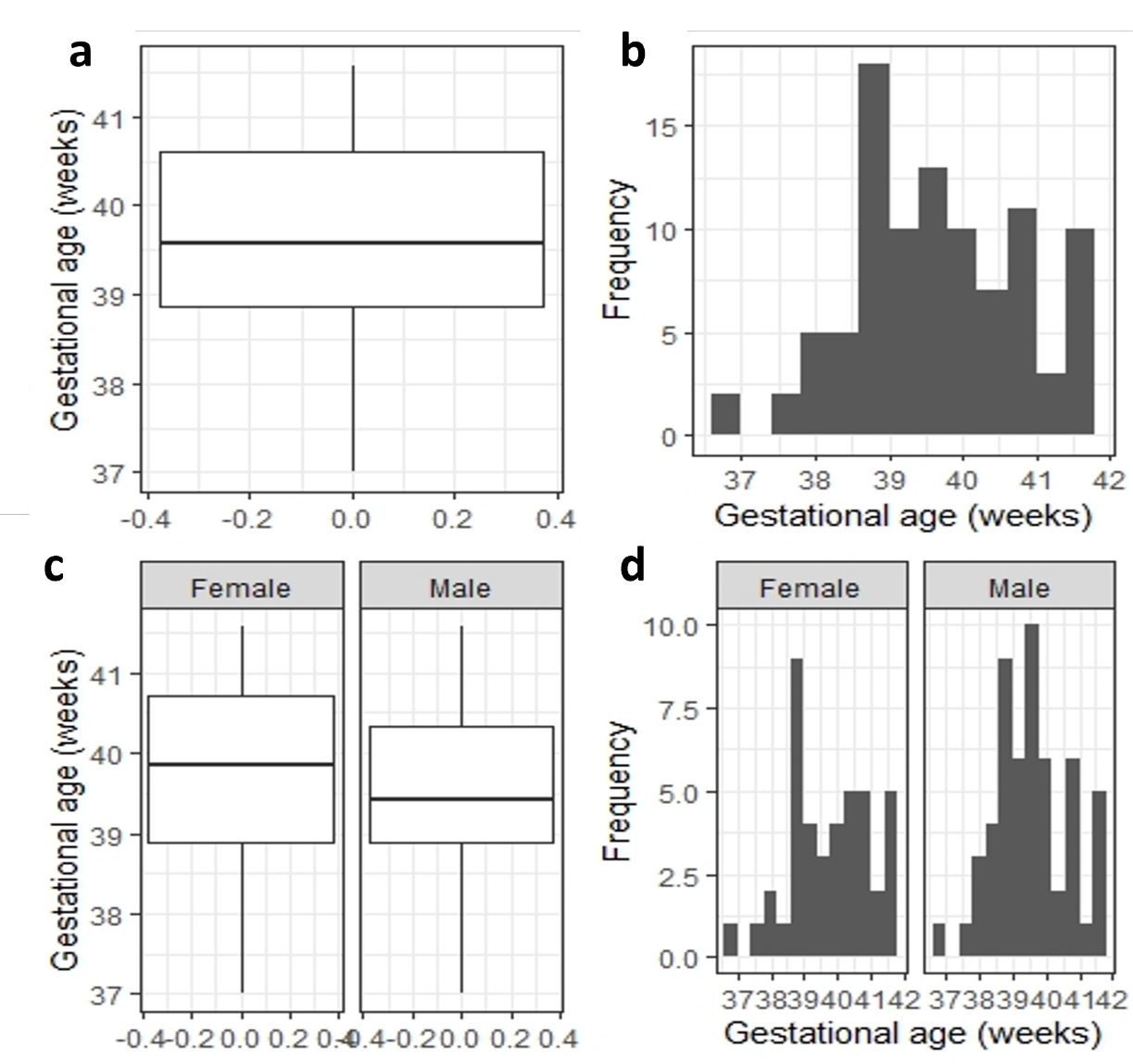
**

**Supplementary figure SF1** – Gestational age distribution in the sample (**a** and **b** panels) and by sex (**c** and **d** panels).

**Supplementary table ST1** – Core microbiome.

| **Phylum** | **Class** | **Order** | **Family** | **Genus** | **Mean relative abundance (%)** | **Number of samples in which the taxa were found** |
| --- | --- | --- | --- | --- | --- | --- |
| Actinobacteria | Actinobacteria | Corynebacteriales | *Corynebacteriaceae* | *Corynebacterium 1* | 1.108 | 54 |
|  |  | Propionibacteriales | *Propionibacteriaceae* | *Cutibacterium* | 0.818 | 60 |
| Bacteroidetes | Bacteroidia | Flavobacteriales | *Crocinitomicaceae* | *Fluviicola* | 0.854 | 56 |
|  |  |  | *Flavobacteriaceae* | *Flavobacterium* | 16.211 | 92 |
| Firmicutes | Bacilli | Bacillales | *Staphylococcaceae* | *Staphylococcus* | 5.128 | 68 |
|  |  | Lactobacillales | *Streptococcaceae* | *Streptococcus* | 1.763 | 59 |
| Proteobacteria | Gammaproteobacteria | Betaproteobacteriales | *Burkholderiaceae* | *Limnohabitans* | 0.833 | 58 |
|  |  | Enterobacteriales | *Enterobacteriaceae* | *Escherichia-Shigella* | 14.100 | 78 |
|  |  | Pseudomonadales | *Moraxellaceae* | *Acinetobacter* | 1.554 | 66 |
| Other phyla | Other classes | Other orders | Other families | Other genera | 57.632 | - |

In **Supplementary table ST1** the core microbiome at genus level is reported with the corresponding higher taxonomic levels.

**Supplementary figure SF2** – Bar plot of predicted pathways’ composition for all samples at level 3. Samples (MEC01-MEC96) are reported on x-axis, while the y-axis indicates the average percentual relative abundance (RA) of each pathway.

**Supplementary figure SF3** – Bar plot of predicted pathways’ composition for all samples at level 2. Samples (MEC01-MEC96) are reported on x-axis, while the y-axis indicates the average percentual relative abundance of each pathway.

**Supplementary table ST2** – List of the predicted microbial pathways.

| **Level 3** | **Level 2** | **Level 1** | **KO pathway** |
| --- | --- | --- | --- |
| Organismal Systems | Sensory system | Inflammatory mediator regulation of TRP channels | ko04750 |
| Organismal Systems | Nervous system | Synaptic vesicle cycle | ko04721 |
| Organismal Systems | Nervous system | Serotonergic synapse | ko04726 |
| Organismal Systems | Nervous system | Retrograde endocannabinoid signaling | ko04723 |
| Organismal Systems | Nervous system | Long-term potentiation | ko04720 |
| Organismal Systems | Nervous system | Long-term depression | ko04730 |
| Organismal Systems | Nervous system | Glutamatergic synapse | ko04724 |
| Organismal Systems | Nervous system | GABAergic synapse | ko04727 |
| Organismal Systems | Nervous system | Dopaminergic synapse | ko04728 |
| Organismal Systems | Nervous system | Cholinergic synapse | ko04725 |
| Organismal Systems | Immune system | Th17 cell differentiation | ko04659 |
| Organismal Systems | Immune system | RIG-I-like receptor signaling pathway | ko04622 |
| Organismal Systems | Immune system | Platelet activation | ko04611 |
| Organismal Systems | Immune system | NOD-like receptor signaling pathway | ko04621 |
| Organismal Systems | Immune system | IL-17 signaling pathway | ko04657 |
| Organismal Systems | Immune system | Hematopoietic cell lineage | ko04640 |
| Organismal Systems | Immune system | Fc gamma R-mediated phagocytosis | ko04666 |
| Organismal Systems | Immune system | C-type lectin receptor signaling pathway | ko04625 |
| Organismal Systems | Immune system | Complement and coagulation cascades | ko04610 |
| Organismal Systems | Immune system | Antigen processing and presentation | ko04612 |
| Organismal Systems | Excretory system | Vasopressin-regulated water reabsorption | ko04962 |
| Organismal Systems | Excretory system | Proximal tubule bicarbonate reclamation | ko04964 |
| Organismal Systems | Excretory system | Endocrine and other factor-regulated calcium reabsorption | ko04961 |
| Organismal Systems | Excretory system | Aldosterone-regulated sodium reabsorption | ko04960 |
| Organismal Systems | Environmental adaptation | Thermogenesis | ko04714 |
| Organismal Systems | Environmental adaptation | Plant-pathogen interaction | ko04626 |
| Organismal Systems | Endocrine system | Thyroid hormone synthesis | ko04918 |
| Organismal Systems | Endocrine system | Thyroid hormone signaling pathway | ko04919 |
| Organismal Systems | Endocrine system | Renin-angiotensin system | ko04614 |
| Organismal Systems | Endocrine system | Renin secretion | ko04924 |
| Organismal Systems | Endocrine system | Relaxin signaling pathway | ko04926 |
| Organismal Systems | Endocrine system | Regulation of lipolysis in adipocytes | ko04923 |
| Organismal Systems | Endocrine system | Prolactin signaling pathway | ko04917 |
| Organismal Systems | Endocrine system | Progesterone-mediated oocyte maturation | ko04914 |
| Organismal Systems | Endocrine system | PPAR signaling pathway | ko03320 |
| Organismal Systems | Endocrine system | Oxytocin signaling pathway | ko04921 |
| Organismal Systems | Endocrine system | Ovarian steroidogenesis | ko04913 |
| Organismal Systems | Endocrine system | Melanogenesis | ko04916 |
| Organismal Systems | Endocrine system | Insulin signaling pathway | ko04910 |
| Organismal Systems | Endocrine system | Insulin secretion | ko04911 |
| Organismal Systems | Endocrine system | GnRH signaling pathway | ko04912 |
| Organismal Systems | Endocrine system | Glucagon signaling pathway | ko04922 |
| Organismal Systems | Endocrine system | Estrogen signaling pathway | ko04915 |
| Organismal Systems | Endocrine system | Aldosterone synthesis and secretion | ko04925 |
| Organismal Systems | Endocrine system | Adipocytokine signaling pathway | ko04920 |
| Organismal Systems | Digestive system | Salivary secretion | ko04970 |
| Organismal Systems | Digestive system | Protein digestion and absorption | ko04974 |
| Organismal Systems | Digestive system | Pancreatic secretion | ko04972 |
| Organismal Systems | Digestive system | Mineral absorption | ko04978 |
| Organismal Systems | Digestive system | Gastric acid secretion | ko04971 |
| Organismal Systems | Digestive system | Cholesterol metabolism | ko04979 |
| Organismal Systems | Digestive system | Carbohydrate digestion and absorption | ko04973 |
| Organismal Systems | Digestive system | Bile secretion | ko04976 |
| Organismal Systems | Development | Osteoclast differentiation | ko04380 |
| Organismal Systems | Circulatory system | Vascular smooth muscle contraction | ko04270 |
| Organismal Systems | Circulatory system | Cardiac muscle contraction | ko04260 |
| Organismal Systems | Circulatory system | Adrenergic signaling in cardiomyocytes | ko04261 |
| Organismal Systems | Aging | Longevity regulating pathway - worm | ko04212 |
| Organismal Systems | Aging | Longevity regulating pathway - multiple species | ko04213 |
| Organismal Systems | Aging | Longevity regulating pathway | ko04211 |
| Metabolism | Xenobiotics biodegradation and metabolism | Xylene degradation | ko00622 |
| Metabolism | Xenobiotics biodegradation and metabolism | Toluene degradation | ko00623 |
| Metabolism | Xenobiotics biodegradation and metabolism | Styrene degradation | ko00643 |
| Metabolism | Xenobiotics biodegradation and metabolism | Steroid degradation | ko00984 |
| Metabolism | Xenobiotics biodegradation and metabolism | Polycyclic aromatic hydrocarbon degradation | ko00624 |
| Metabolism | Xenobiotics biodegradation and metabolism | Nitrotoluene degradation | ko00633 |
| Metabolism | Xenobiotics biodegradation and metabolism | Naphthalene degradation | ko00626 |
| Metabolism | Xenobiotics biodegradation and metabolism | Metabolism of xenobiotics by cytochrome P450 | ko00980 |
| Metabolism | Xenobiotics biodegradation and metabolism | Furfural degradation | ko00365 |
| Metabolism | Xenobiotics biodegradation and metabolism | Fluorobenzoate degradation | ko00364 |
| Metabolism | Xenobiotics biodegradation and metabolism | Ethylbenzene degradation | ko00642 |
| Metabolism | Xenobiotics biodegradation and metabolism | Drug metabolism - other enzymes | ko00983 |
| Metabolism | Xenobiotics biodegradation and metabolism | Drug metabolism - cytochrome P450 | ko00982 |
| Metabolism | Xenobiotics biodegradation and metabolism | Dioxin degradation | ko00621 |
| Metabolism | Xenobiotics biodegradation and metabolism | Chlorocyclohexane and chlorobenzene degradation | ko00361 |
| Metabolism | Xenobiotics biodegradation and metabolism | Chloroalkane and chloroalkene degradation | ko00625 |
| Metabolism | Xenobiotics biodegradation and metabolism | Caprolactam degradation | ko00930 |
| Metabolism | Xenobiotics biodegradation and metabolism | Bisphenol degradation | ko00363 |
| Metabolism | Xenobiotics biodegradation and metabolism | Benzoate degradation | ko00362 |
| Metabolism | Xenobiotics biodegradation and metabolism | Atrazine degradation | ko00791 |
| Metabolism | Xenobiotics biodegradation and metabolism | Aminobenzoate degradation | ko00627 |
| Metabolism | Nucleotide metabolism | Pyrimidine metabolism | ko00240 |
| Metabolism | Nucleotide metabolism | Purine metabolism | ko00230 |
| Metabolism | Metabolism of terpenoids and polyketides | Zeatin biosynthesis | ko00908 |
| Metabolism | Metabolism of terpenoids and polyketides | Type I polyketide structures | ko01052 |
| Metabolism | Metabolism of terpenoids and polyketides | Tetracycline biosynthesis | ko00253 |
| Metabolism | Metabolism of terpenoids and polyketides | Terpenoid backbone biosynthesis | ko00900 |
| Metabolism | Metabolism of terpenoids and polyketides | Sesquiterpenoid and triterpenoid biosynthesis | ko00909 |
| Metabolism | Metabolism of terpenoids and polyketides | Polyketide sugar unit biosynthesis | ko00523 |
| Metabolism | Metabolism of terpenoids and polyketides | Nonribosomal peptide structures | ko01054 |
| Metabolism | Metabolism of terpenoids and polyketides | Monoterpenoid biosynthesis | ko00902 |
| Metabolism | Metabolism of terpenoids and polyketides | Limonene and pinene degradation | ko00903 |
| Metabolism | Metabolism of terpenoids and polyketides | Insect hormone biosynthesis | ko00981 |
| Metabolism | Metabolism of terpenoids and polyketides | Geraniol degradation | ko00281 |
| Metabolism | Metabolism of terpenoids and polyketides | Carotenoid biosynthesis | ko00906 |
| Metabolism | Metabolism of terpenoids and polyketides | Biosynthesis of vancomycin group antibiotics | ko01055 |
| Metabolism | Metabolism of terpenoids and polyketides | Biosynthesis of type II polyketide products | ko01057 |
| Metabolism | Metabolism of terpenoids and polyketides | Biosynthesis of type II polyketide backbone | ko01056 |
| Metabolism | Metabolism of terpenoids and polyketides | Biosynthesis of siderophore group nonribosomal peptides | ko01053 |
| Metabolism | Metabolism of terpenoids and polyketides | Biosynthesis of enediyne antibiotics | ko01059 |
| Metabolism | Metabolism of terpenoids and polyketides | Biosynthesis of ansamycins | ko01051 |
| Metabolism | Metabolism of other amino acids | Taurine and hypotaurine metabolism | ko00430 |
| Metabolism | Metabolism of other amino acids | Selenocompound metabolism | ko00450 |
| Metabolism | Metabolism of other amino acids | Phosphonate and phosphinate metabolism | ko00440 |
| Metabolism | Metabolism of other amino acids | Glutathione metabolism | ko00480 |
| Metabolism | Metabolism of other amino acids | D-Glutamine and D-glutamate metabolism | ko00471 |
| Metabolism | Metabolism of other amino acids | D-Arginine and D-ornithine metabolism | ko00472 |
| Metabolism | Metabolism of other amino acids | D-Alanine metabolism | ko00473 |
| Metabolism | Metabolism of other amino acids | Cyanoamino acid metabolism | ko00460 |
| Metabolism | Metabolism of other amino acids | beta-Alanine metabolism | ko00410 |
| Metabolism | Metabolism of cofactors and vitamins | Vitamin B6 metabolism | ko00750 |
| Metabolism | Metabolism of cofactors and vitamins | Ubiquinone and other terpenoid-quinone biosynthesis | ko00130 |
| Metabolism | Metabolism of cofactors and vitamins | Thiamine metabolism | ko00730 |
| Metabolism | Metabolism of cofactors and vitamins | Riboflavin metabolism | ko00740 |
| Metabolism | Metabolism of cofactors and vitamins | Retinol metabolism | ko00830 |
| Metabolism | Metabolism of cofactors and vitamins | Porphyrin and chlorophyll metabolism | ko00860 |
| Metabolism | Metabolism of cofactors and vitamins | Pantothenate and CoA biosynthesis | ko00770 |
| Metabolism | Metabolism of cofactors and vitamins | One carbon pool by folate | ko00670 |
| Metabolism | Metabolism of cofactors and vitamins | Nicotinate and nicotinamide metabolism | ko00760 |
| Metabolism | Metabolism of cofactors and vitamins | Lipoic acid metabolism | ko00785 |
| Metabolism | Metabolism of cofactors and vitamins | Folate biosynthesis | ko00790 |
| Metabolism | Metabolism of cofactors and vitamins | Biotin metabolism | ko00780 |
| Metabolism | Lipid metabolism | Synthesis and degradation of ketone bodies | ko00072 |
| Metabolism | Lipid metabolism | Steroid hormone biosynthesis | ko00140 |
| Metabolism | Lipid metabolism | Steroid biosynthesis | ko00100 |
| Metabolism | Lipid metabolism | Sphingolipid metabolism | ko00600 |
| Metabolism | Lipid metabolism | Secondary bile acid biosynthesis | ko00121 |
| Metabolism | Lipid metabolism | Primary bile acid biosynthesis | ko00120 |
| Metabolism | Lipid metabolism | Linoleic acid metabolism | ko00591 |
| Metabolism | Lipid metabolism | Glycerophospholipid metabolism | ko00564 |
| Metabolism | Lipid metabolism | Glycerolipid metabolism | ko00561 |
| Metabolism | Lipid metabolism | Fatty acid elongation | ko00062 |
| Metabolism | Lipid metabolism | Fatty acid degradation | ko00071 |
| Metabolism | Lipid metabolism | Fatty acid biosynthesis | ko00061 |
| Metabolism | Lipid metabolism | Ether lipid metabolism | ko00565 |
| Metabolism | Lipid metabolism | Biosynthesis of unsaturated fatty acids | ko01040 |
| Metabolism | Lipid metabolism | Arachidonic acid metabolism | ko00590 |
| Metabolism | Lipid metabolism | alpha-Linolenic acid metabolism | ko00592 |
| Metabolism | Glycan biosynthesis and metabolism | Various types of N-glycan biosynthesis | ko00513 |
| Metabolism | Glycan biosynthesis and metabolism | Peptidoglycan biosynthesis | ko00550 |
| Metabolism | Glycan biosynthesis and metabolism | Other types of O-glycan biosynthesis | ko00514 |
| Metabolism | Glycan biosynthesis and metabolism | Other glycan degradation | ko00511 |
| Metabolism | Glycan biosynthesis and metabolism | N-Glycan biosynthesis | ko00510 |
| Metabolism | Glycan biosynthesis and metabolism | Mannose type O-glycan biosynthesis | ko00515 |
| Metabolism | Glycan biosynthesis and metabolism | Lipopolysaccharide biosynthesis | ko00540 |
| Metabolism | Glycan biosynthesis and metabolism | Lipoarabinomannan (LAM) biosynthesis | ko00571 |
| Metabolism | Glycan biosynthesis and metabolism | Glycosylphosphatidylinositol (GPI)-anchor biosynthesis | ko00563 |
| Metabolism | Glycan biosynthesis and metabolism | Glycosphingolipid biosynthesis - lacto and neolacto series | ko00601 |
| Metabolism | Glycan biosynthesis and metabolism | Glycosphingolipid biosynthesis - globo and isoglobo series | ko00603 |
| Metabolism | Glycan biosynthesis and metabolism | Glycosphingolipid biosynthesis - ganglio series | ko00604 |
| Metabolism | Glycan biosynthesis and metabolism | Glycosaminoglycan degradation | ko00531 |
| Metabolism | Glycan biosynthesis and metabolism | Glycosaminoglycan biosynthesis - heparan sulfate / heparin | ko00534 |
| Metabolism | Glycan biosynthesis and metabolism | Glycosaminoglycan biosynthesis - chondroitin sulfate / dermatan sulfate | ko00532 |
| Metabolism | Global and overview maps | Microbial metabolism in diverse environments | ko01120 |
| Metabolism | Global and overview maps | Metabolic pathways | ko01100 |
| Metabolism | Global and overview maps | Fatty acid metabolism | ko01212 |
| Metabolism | Global and overview maps | Degradation of aromatic compounds | ko01220 |
| Metabolism | Global and overview maps | Carbon metabolism | ko01200 |
| Metabolism | Global and overview maps | Biosynthesis of secondary metabolites | ko01110 |
| Metabolism | Global and overview maps | Biosynthesis of antibiotics | ko01130 |
| Metabolism | Global and overview maps | Biosynthesis of amino acids | ko01230 |
| Metabolism | Global and overview maps | 2-Oxocarboxylic acid metabolism | ko01210 |
| Metabolism | Energy metabolism | Sulfur metabolism | ko00920 |
| Metabolism | Energy metabolism | Photosynthesis - antenna proteins | ko00196 |
| Metabolism | Energy metabolism | Photosynthesis | ko00195 |
| Metabolism | Energy metabolism | Oxidative phosphorylation | ko00190 |
| Metabolism | Energy metabolism | Nitrogen metabolism | ko00910 |
| Metabolism | Energy metabolism | Methane metabolism | ko00680 |
| Metabolism | Energy metabolism | Carbon fixation pathways in prokaryotes | ko00720 |
| Metabolism | Energy metabolism | Carbon fixation in photosynthetic organisms | ko00710 |
| Metabolism | Chemical structure transformation maps | Biosynthesis of terpenoids and steroids | ko01062 |
| Metabolism | Carbohydrate metabolism | Starch and sucrose metabolism | ko00500 |
| Metabolism | Carbohydrate metabolism | Pyruvate metabolism | ko00620 |
| Metabolism | Carbohydrate metabolism | Propanoate metabolism | ko00640 |
| Metabolism | Carbohydrate metabolism | Pentose phosphate pathway | ko00030 |
| Metabolism | Carbohydrate metabolism | Pentose and glucuronate interconversions | ko00040 |
| Metabolism | Carbohydrate metabolism | Inositol phosphate metabolism | ko00562 |
| Metabolism | Carbohydrate metabolism | Glyoxylate and dicarboxylate metabolism | ko00630 |
| Metabolism | Carbohydrate metabolism | Glycolysis | ko00010 |
| Metabolism | Carbohydrate metabolism | Galactose metabolism | ko00052 |
| Metabolism | Carbohydrate metabolism | Fructose and mannose metabolism | ko00051 |
| Metabolism | Carbohydrate metabolism | Citrate cycle (TCA cycle) | ko00020 |
| Metabolism | Carbohydrate metabolism | C5-Branched dibasic acid metabolism | ko00660 |
| Metabolism | Carbohydrate metabolism | Butanoate metabolism | ko00650 |
| Metabolism | Carbohydrate metabolism | Ascorbate and aldarate metabolism | ko00053 |
| Metabolism | Carbohydrate metabolism | Amino sugar and nucleotide sugar metabolism | ko00520 |
| Metabolism | Biosynthesis of other secondary metabolites | Streptomycin biosynthesis | ko00521 |
| Metabolism | Biosynthesis of other secondary metabolites | Staurosporine biosynthesis | ko00404 |
| Metabolism | Biosynthesis of other secondary metabolites | Prodigiosin biosynthesis | ko00333 |
| Metabolism | Biosynthesis of other secondary metabolites | Phenylpropanoid biosynthesis | ko00940 |
| Metabolism | Biosynthesis of other secondary metabolites | Phenazine biosynthesis | ko00405 |
| Metabolism | Biosynthesis of other secondary metabolites | Penicillin and cephalosporin biosynthesis | ko00311 |
| Metabolism | Biosynthesis of other secondary metabolites | Novobiocin biosynthesis | ko00401 |
| Metabolism | Biosynthesis of other secondary metabolites | Monobactam biosynthesis | ko00261 |
| Metabolism | Biosynthesis of other secondary metabolites | Isoquinoline alkaloid biosynthesis | ko00950 |
| Metabolism | Biosynthesis of other secondary metabolites | Isoflavonoid biosynthesis | ko00943 |
| Metabolism | Biosynthesis of other secondary metabolites | Indole alkaloid biosynthesis | ko00901 |
| Metabolism | Biosynthesis of other secondary metabolites | Glucosinolate biosynthesis | ko00966 |
| Metabolism | Biosynthesis of other secondary metabolites | Flavonoid biosynthesis | ko00941 |
| Metabolism | Biosynthesis of other secondary metabolites | Flavone and flavonol biosynthesis | ko00944 |
| Metabolism | Biosynthesis of other secondary metabolites | Clavulanic acid biosynthesis | ko00331 |
| Metabolism | Biosynthesis of other secondary metabolites | Carbapenem biosynthesis | ko00332 |
| Metabolism | Biosynthesis of other secondary metabolites | Caffeine metabolism | ko00232 |
| Metabolism | Biosynthesis of other secondary metabolites | Betalain biosynthesis | ko00965 |
| Metabolism | Biosynthesis of other secondary metabolites | Acarbose and validamycin biosynthesis | ko00525 |
| Metabolism | Amino acid metabolism | Tyrosine metabolism | ko00350 |
| Metabolism | Amino acid metabolism | Tryptophan metabolism | ko00380 |
| Metabolism | Amino acid metabolism | Phenylalanine metabolism | ko00360 |
| Metabolism | Amino acid metabolism | Lysine degradation | ko00310 |
| Metabolism | Amino acid metabolism | Lysine biosynthesis | ko00300 |
| Metabolism | Amino acid metabolism | Histidine metabolism | ko00340 |
| Metabolism | Amino acid metabolism | Cysteine and methionine metabolism | ko00270 |
| Metabolism | Amino acid metabolism | Arginine biosynthesis | ko00220 |
| Metabolism | Amino acid metabolism | Arginine and proline metabolism | ko00330 |
| Human Diseases | Substance dependence | Cocaine addiction | ko05030 |
| Human Diseases | Substance dependence | Amphetamine addiction | ko05031 |
| Human Diseases | Substance dependence | Alcoholism | ko05034 |
| Human Diseases | Neurodegenerative diseases | Prion diseases | ko05020 |
| Human Diseases | Neurodegenerative diseases | Parkinson's disease | ko05012 |
| Human Diseases | Neurodegenerative diseases | Huntington's disease | ko05016 |
| Human Diseases | Neurodegenerative diseases | Amyotrophic lateral sclerosis (ALS) | ko05014 |
| Human Diseases | Neurodegenerative diseases | Alzheimer's disease | ko05010 |
| Human Diseases | Infectious diseases: Viral | Measles | ko05162 |
| Human Diseases | Infectious diseases: Viral | Kaposi's sarcoma-associated herpesvirus infection | ko05167 |
| Human Diseases | Infectious diseases: Viral | Influenza A | ko05164 |
| Human Diseases | Infectious diseases: Viral | Human papillomavirus infection | ko05165 |
| Human Diseases | Infectious diseases: Viral | Human cytomegalovirus infection | ko05163 |
| Human Diseases | Infectious diseases: Viral | HTLV-I infection | ko05166 |
| Human Diseases | Infectious diseases: Viral | Herpes simplex infection | ko05168 |
| Human Diseases | Infectious diseases: Viral | Hepatitis C | ko05160 |
| Human Diseases | Infectious diseases: Viral | Hepatitis B | ko05161 |
| Human Diseases | Infectious diseases: Viral | Epstein-Barr virus infection | ko05169 |
| Human Diseases | Infectious diseases: Parasitic | Toxoplasmosis | ko05145 |
| Human Diseases | Infectious diseases: Parasitic | Leishmaniasis | ko05140 |
| Human Diseases | Infectious diseases: Parasitic | Chagas disease (American trypanosomiasis) | ko05142 |
| Human Diseases | Infectious diseases: Parasitic | Amoebiasis | ko05146 |
| Human Diseases | Infectious diseases: Parasitic | African trypanosomiasis | ko05143 |
| Human Diseases | Infectious diseases: Bacterial | Vibrio cholerae infection | ko05110 |
| Human Diseases | Infectious diseases: Bacterial | Tuberculosis | ko05152 |
| Human Diseases | Infectious diseases: Bacterial | Staphylococcus aureus infection | ko05150 |
| Human Diseases | Infectious diseases: Bacterial | Shigellosis | ko05131 |
| Human Diseases | Infectious diseases: Bacterial | Salmonella infection | ko05132 |
| Human Diseases | Infectious diseases: Bacterial | Pertussis | ko05133 |
| Human Diseases | Infectious diseases: Bacterial | Pathogenic Escherichia coli infection | ko05130 |
| Human Diseases | Infectious diseases: Bacterial | Legionellosis | ko05134 |
| Human Diseases | Infectious diseases: Bacterial | Epithelial cell signaling in Helicobacter pylori infection | ko05120 |
| Human Diseases | Infectious diseases: Bacterial | Bacterial invasion of epithelial cells | ko05100 |
| Human Diseases | Immune diseases | Systemic lupus erythematosus | ko05322 |
| Human Diseases | Immune diseases | Rheumatoid arthritis | ko05323 |
| Human Diseases | Immune diseases | Primary immunodeficiency | ko05340 |
| Human Diseases | Endocrine and metabolic diseases | Type II diabetes mellitus | ko04930 |
| Human Diseases | Endocrine and metabolic diseases | Type I diabetes mellitus | ko04940 |
| Human Diseases | Endocrine and metabolic diseases | Non-alcoholic fatty liver disease (NAFLD) | ko04932 |
| Human Diseases | Endocrine and metabolic diseases | Insulin resistance | ko04931 |
| Human Diseases | Endocrine and metabolic diseases | Cushing's syndrome | ko04934 |
| Human Diseases | Endocrine and metabolic diseases | AGE-RAGE signaling pathway in diabetic complications | ko04933 |
| Human Diseases | Drug resistance: Antineoplastic | Platinum drug resistance | ko01524 |
| Human Diseases | Drug resistance: Antineoplastic | EGFR tyrosine kinase inhibitor resistance | ko01521 |
| Human Diseases | Drug resistance: Antineoplastic | Antifolate resistance | ko01523 |
| Human Diseases | Drug resistance: Antimicrobial | Vancomycin resistance | ko01502 |
| Human Diseases | Drug resistance: Antimicrobial | Cationic antimicrobial peptide (CAMP) resistance | ko01503 |
| Human Diseases | Drug resistance: Antimicrobial | beta-Lactam resistance | ko01501 |
| Human Diseases | Cardiovascular diseases | Viral myocarditis | ko05416 |
| Human Diseases | Cardiovascular diseases | Hypertrophic cardiomyopathy (HCM) | ko05410 |
| Human Diseases | Cardiovascular diseases | Fluid shear stress and atherosclerosis | ko05418 |
| Human Diseases | Cardiovascular diseases | Dilated cardiomyopathy (DCM) | ko05414 |
| Human Diseases | Cancers: Specific types | Small cell lung cancer | ko05222 |
| Human Diseases | Cancers: Specific types | Renal cell carcinoma | ko05211 |
| Human Diseases | Cancers: Specific types | Prostate cancer | ko05215 |
| Human Diseases | Cancers: Specific types | Hepatocellular carcinoma | ko05225 |
| Human Diseases | Cancers: Specific types | Colorectal cancer | ko05210 |
| Human Diseases | Cancers: Specific types | Chronic myeloid leukemia | ko05220 |
| Human Diseases | Cancers: Specific types | Bladder cancer | ko05219 |
| Human Diseases | Cancers: Overview | Viral carcinogenesis | ko05203 |
| Human Diseases | Cancers: Overview | Proteoglycans in cancer | ko05205 |
| Human Diseases | Cancers: Overview | Pathways in cancer | ko05200 |
| Human Diseases | Cancers: Overview | MicroRNAs in cancer | ko05206 |
| Human Diseases | Cancers: Overview | Choline metabolism in cancer | ko05231 |
| Human Diseases | Cancers: Overview | Chemical carcinogenesis | ko05204 |
| Human Diseases | Cancers: Overview | Central carbon metabolism in cancer | ko05230 |
| Genetic Information Processing | Translation | RNA transport | ko03013 |
| Genetic Information Processing | Translation | Ribosome biogenesis in eukaryotes | ko03008 |
| Genetic Information Processing | Translation | Ribosome | ko03010 |
| Genetic Information Processing | Translation | mRNA surveillance pathway | ko03015 |
| Genetic Information Processing | Translation | Aminoacyl-tRNA biosynthesis | ko00970 |
| Genetic Information Processing | Transcription | Spliceosome | ko03040 |
| Genetic Information Processing | Transcription | RNA polymerase | ko03020 |
| Genetic Information Processing | Transcription | Basal transcription factors | ko03022 |
| Genetic Information Processing | Replication and repair | Nucleotide excision repair | ko03420 |
| Genetic Information Processing | Replication and repair | Non-homologous end-joining | ko03450 |
| Genetic Information Processing | Replication and repair | Mismatch repair | ko03430 |
| Genetic Information Processing | Replication and repair | Homologous recombination | ko03440 |
| Genetic Information Processing | Replication and repair | Fanconi anemia pathway | ko03460 |
| Genetic Information Processing | Replication and repair | DNA replication | ko03030 |
| Genetic Information Processing | Replication and repair | Base excision repair | ko03410 |
| Environmental Information Processing | Signaling molecules and interaction | Neuroactive ligand-receptor interaction | ko04080 |
| Environmental Information Processing | Signaling molecules and interaction | ECM-receptor interaction | ko04512 |
| Environmental Information Processing | Signal transduction | Wnt signaling pathway | ko04310 |
| Environmental Information Processing | Signal transduction | VEGF signaling pathway | ko04370 |
| Environmental Information Processing | Signal transduction | Two-component system | ko02020 |
| Environmental Information Processing | Signal transduction | TNF signaling pathway | ko04668 |
| Environmental Information Processing | Signal transduction | TGF-beta signaling pathway | ko04350 |
| Environmental Information Processing | Signal transduction | Sphingolipid signaling pathway | ko04071 |
| Environmental Information Processing | Signal transduction | Ras signaling pathway | ko04014 |
| Environmental Information Processing | Signal transduction | PI3K-Akt signaling pathway | ko04151 |
| Environmental Information Processing | Signal transduction | Phospholipase D signaling pathway | ko04072 |
| Environmental Information Processing | Signal transduction | Phosphatidylinositol signaling system | ko04070 |
| Environmental Information Processing | Signal transduction | Notch signaling pathway | ko04330 |
| Environmental Information Processing | Signal transduction | NF-kappa B signaling pathway | ko04064 |
| Environmental Information Processing | Signal transduction | mTOR signaling pathway | ko04150 |
| Environmental Information Processing | Signal transduction | MAPK signaling pathway - yeast | ko04011 |
| Environmental Information Processing | Signal transduction | MAPK signaling pathway - plant | ko04016 |
| Environmental Information Processing | Signal transduction | MAPK signaling pathway - fly | ko04013 |
| Environmental Information Processing | Signal transduction | Hippo signaling pathway - fly | ko04391 |
| Environmental Information Processing | Signal transduction | Hippo signaling pathway | ko04390 |
| Environmental Information Processing | Signal transduction | HIF-1 signaling pathway | ko04066 |
| Environmental Information Processing | Signal transduction | FoxO signaling pathway | ko04068 |
| Environmental Information Processing | Signal transduction | cGMP-PKG signaling pathway | ko04022 |
| Environmental Information Processing | Signal transduction | cAMP signaling pathway | ko04024 |
| Environmental Information Processing | Signal transduction | Calcium signaling pathway | ko04020 |
| Environmental Information Processing | Signal transduction | Apelin signaling pathway | ko04371 |
| Environmental Information Processing | Signal transduction | AMPK signaling pathway | ko04152 |
| Environmental Information Processing | Membrane transport | Phosphotransferase system (PTS) | ko02060 |
| Environmental Information Processing | Membrane transport | Bacterial secretion system | ko03070 |
| Environmental Information Processing | Membrane transport | ABC transporters | ko02010 |
| Cellular Processes | Transport and catabolism | Phagosome | ko04145 |
| Cellular Processes | Transport and catabolism | Peroxisome | ko04146 |
| Cellular Processes | Transport and catabolism | Lysosome | ko04142 |
| Cellular Processes | Transport and catabolism | Endocytosis | ko04144 |
| Cellular Processes | Transport and catabolism | Autophagy - yeast | ko04138 |
| Cellular Processes | Transport and catabolism | Autophagy - animal | ko04140 |
| Cellular Processes | Cellular community - prokaryotes | Quorum sensing | ko02024 |
| Cellular Processes | Cellular community - prokaryotes | Biofilm formation - Vibrio cholerae | ko05111 |
| Cellular Processes | Cellular community - prokaryotes | Biofilm formation - Pseudomonas aeruginosa | ko02025 |
| Cellular Processes | Cellular community - prokaryotes | Biofilm formation - Escherichia coli | ko02026 |
| Cellular Processes | Cellular community - eukaryotes | Tight junction | ko04530 |
| Cellular Processes | Cellular community - eukaryotes | Focal adhesion | ko04510 |
| Cellular Processes | Cell motility | Regulation of actin cytoskeleton | ko04810 |
| Cellular Processes | Cell motility | Flagellar assembly | ko02040 |
| Cellular Processes | Cell motility | Bacterial chemotaxis | ko02030 |
| Cellular Processes | Cell growth and death | p53 signaling pathway | ko04115 |
| Cellular Processes | Cell growth and death | Oocyte meiosis | ko04114 |
| Cellular Processes | Cell growth and death | Necroptosis | ko04217 |
| Cellular Processes | Cell growth and death | Meiosis - yeast | ko04113 |
| Cellular Processes | Cell growth and death | Ferroptosis | ko04216 |
| Cellular Processes | Cell growth and death | Cellular senescence | ko04218 |
| Cellular Processes | Cell growth and death | Cell cycle - yeast | ko04111 |
| Cellular Processes | Cell growth and death | Cell cycle - Caulobacter | ko04112 |
| Cellular Processes | Cell growth and death | Cell cycle | ko04110 |
| Cellular Processes | Cell growth and death | Apoptosis - multiple species | ko04215 |
| Cellular Processes | Cell growth and death | Apoptosis - fly | ko04214 |
| Cellular Processes | Cell growth and death | Apoptosis | ko04210 |
| - | Folding | Sulfur relay system | ko04122 |
| - | Folding | RNA degradation | ko03018 |
| - | Folding | Protein processing in endoplasmic reticulum | ko04141 |
| - | Folding | Protein export | ko03060 |
| - | Folding | Proteasome | ko03050 |
| - | - | Valine | ko00280 |
| - | - | Valine | ko00290 |
| - | - | Tropane | ko00960 |
| - | - | Stilbenoid | ko00945 |
| - | - | Phenylalanine | ko00400 |
| - | - | Parathyroid hormone synthesis | ko04928 |
| - | - | Neomycin | ko00524 |
| - | - | Glycine | ko00260 |
| - | - | Cutin | ko00073 |
| - | - | Biosynthesis of 12- | ko00522 |
| - | - | Alanine | ko00250 |


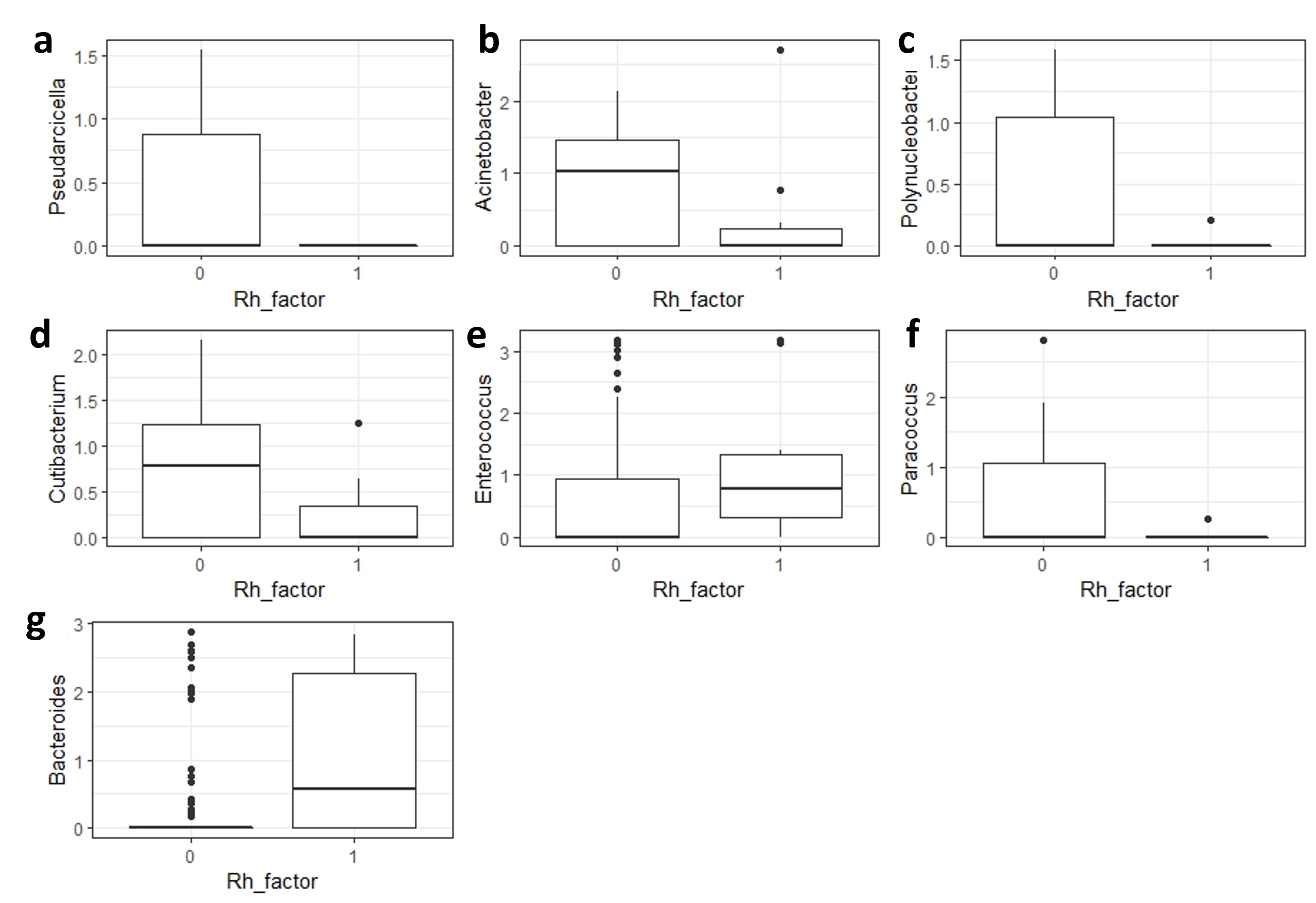


**Supplementary figure SF4** – Differentially abundant taxa by maternal Rh factor with p-value<0.05. In each panel (**a**-**g**), the percentual relative abundance is reported on the y-axis, while the 0 and 1 values on the x-axis stand for Rh positive and negative respectively.

**Supplementary note**

Due to the small amount of starting material and to the presence of many PCR inhibitors in meconium samples, clear DNA bands were hard to visualize. Thus, a second PCR was performed using the amplified DNA from the first PCR as template, with minor modifications of PCR conditions: 6µl of the first PCR were added to 19µl of master mix made of 13.8µl of H_2_O, 2.5µl of 10x Buffer without Mg^2+^, 1µl of 50mM MgSO_4_, 0.5µl of 5mM dNTP mix, 1µl of 1µM PRO341F, 1µl of 1µM PRO805R and 0.2 µl of Platinum Polymerase (final concentration of 2U/rxn).

**Supplementary table ST3** – Primers for 16S gene amplification.

| **Primer** | **Sequence** |
| --- | --- |
| PRO341F | 5’-TCGTCGGCAGCGTCAGATGTGTATAAGAGACAGCCTACGGGNBGCASCAG-3’ |
| PRO805R | 5’-GTCTCGTGGGCTCGGAGATGTGTATAAGAGACAGGACTACNVGGGTATCTAATCC-3’ |

These primers were provided with the adapters (underlined parts) required for the successive sequencing step.
